# Cognitive function and gait speed under normal and dual-task walking among older adults with mild cognitive impairment

**DOI:** 10.1186/1471-2377-14-67

**Published:** 2014-04-01

**Authors:** Takehiko Doi, Hiroyuki Shimada, Hyuma Makizako, Kota Tsutsumimoto, Kazuki Uemura, Yuya Anan, Takao Suzuki

**Affiliations:** 1 Section for Health Promotion, Department for Research and Development to Support Independent Life of Elderly, Center for Gerontology and Social Science, National Center for Geriatrics and Gerontology, 35 Gengo, Morioka, Obu, Aichi 474-8511, Japan; 2 Japan Society for the Promotion of Science, Tokyo, 5-3-1 Koujimachi, Chiyoda, Tokyo 102-8471, Japan; 3 Research Institute, National Center for Geriatrics and Gerontology, 35 Gengo, Morioka, Obu, Aichi 474-8511, Japan

## Abstract

**Background:**

Gait ability and cognitive function are interrelated during both normal walking (NW) and dual-task walking (DTW), and gait ability is thus adversely affected by cognitive impairment in both situations. However, this association is insufficiently understood in people with mild cognitive impairment (MCI). Here, we conducted a study with MCI participants, to examine whether the association depends on walking conditions and MCI subtypes.

**Methods:**

We classified 389 elderly adults into amnestic MCI (n = 191) and non-amnestic MCI (n = 198), assessed their cognitive functions, and administered gait experiments under NW and DTW conditions. Gait ability was defined as gait speed. Five aspects of cognitive function were assessed: processing speed, executive function, working memory, verbal memory, and visual memory.

**Results:**

Regression analysis adjusted for covariates showed a significant association between cognitive functions and gait speed. Processing speed and executive function correlated with gait speed during both NW and DTW (*p* < .05). Gait speed during DTW was also significantly associated with working memory (*p* < .001). Visual memory was associated during NW and DTW, particularly for amnestic MCI participants (*p* < .05).

**Conclusions:**

Our findings support the idea that the association between gait speed and cognitive function depends on walking condition and MCI subtypes. Additional studies are necessary to determine the neural basis for the disruption in gait control in older adults with MCI.

## Background

Dementia is a notable health issue because of its extensive impact on the activities and quality of life of older adults. Given the current absence of disease-modifying treatments, as well as increasing awareness that symptoms develop over many years or even decades, there has been growing interest in early detection and effective strategies for prevention [1]. Mild cognitive impairment (MCI) is considered a clinical characteristic that typifies the prodromal phase of Alzheimer’s disease (AD), the most common type of dementia [2]. Numerous studies have identified a wide range of potentially modifiable risk factors for AD and dementia, including cardiovascular risk factors, psychosocial factors, and health behaviors [1,3]. Gait impairment is a common characteristic in participants with cognitive impairments [4-6] and is a risk factor for developmental MCI and dementia [7,8]. Cognition and gait are thought to be strongly linked, a contention supported by findings from experimental studies using a dual-task paradigm to epidemiology.

Less is known about the relationships between specific cognitive functions and gait in people with MCI, though population studies have been conducted in older adults to examine this issue [9-14]. Prospective studies indicate that lower attention/executive function [9,13] or memory function [9,11] may lead to a decline in gait speed in older adults. Alternatively, a slow gait speed predicts deficits in the cognitive-processing speed [12] or in executive and memory functions [14]. Emerging evidence indicates that cognitive processes related to prefrontal lobe function such as attention and executive function are associated with slower gait and gait instability [15]. However, a consensus regarding the relationship between gait variables and memory deficits in particular has not yet been reached [9-11,14]. Mielke *et al*. has suggested that inconsistencies between studies may be partially due to variation in participant characteristics across studies, ranging from exclusively older adults with normal cognition to mixed participant pools that include those with MCI or AD [14]. In addition, the decline in cognitive function in people with MCI is not uniform, but rather depends on MCI subtype, i.e., amnestic (aMCI) or non-amnestic (naMCI) [2]. Furthermore, subtypes of MCI may potentially have different neuropathologies and courses of conversion, although the dependency of subtypes has not reached consensus [16-20]. Investigating cognitive function in MCI participants requires considering several cognitive function domains as well as these MCI subtypes.

The relationship between cognitive function and gait variables in conditions other than normal walking (NW) is insufficiently understood in people with MCI. Observing how people walk while they perform a secondary attention-demanding task, i.e., a dual-task paradigm, has been used to assess interactions between cognition and gait. Existing population studies have been conducted using both NW and dual-task paradigms with specific conditions [21-23], and gait coordination during dual-task walking (DTW) has been shown to be deteriorated [24,25] and to be associated with reduced executive function [21,22]. Although evidence is scarce, gait variables in older adults with MCI have been shown to be affected in both NW [6] and DTW [26]. Less focus has been given to the association between cognitive function and gait, and no strong conclusions can be drawn because of small MCI sample sizes, non-comprehensive cognitive measurements, or experiments that only examine NW. Thus, a large population study that combines comprehensive cognitive assessments with experiments that include DTW will contribute to a better understanding of the relationship between cognitive function and gait in people with MCI.

Untangling the relationship between early gait disturbances and early cognitive changes may be helpful in identifying older adults who are at risk of mobility decline, falls, and progression to dementia [15]. This study aimed to examine the association between cognitive function and gait speed in older people with MCI, and to examine whether these associations differed depending on walking condition (normal or dual-task) and subtypes of MCI. Gait ability was defined as gait speed following the standard method used in population studies of gait [14].

## Methods

### Participants

The study population and data were in a cohort study. Six hundred and forty-nine participants were selected as a potential study population from a cohort study (Obu Study of Health Promotion for the Elderly [27]) and met the following criteria: over 65 years old, diagnosed with MCI, no specific medical history of cerebrovascular disease, Parkinson’s disease, connective tissue disease, or depression, no severe visual or auditory impairment, no current symptoms of depression (Geriatric Depression Scale ≥6 [28]), not part of other research projects, and not certified to receive support from the Japanese public long-term-care insurance system. As a result of recruitment, 409 responded and after giving their written informed consent 389 people completed the neuropsychological assessments and gait experiments. The ethics committee of the National Center for Geriatrics and Gerontology approved this study.

### MCI criteria

MCI criteria followed those established and revised by Petersen [2], and in particular, participants satisfied the following conditions: 1) memory complaints; 2) objective cognitive decline; 3) intact general cognitive function; and 4) independent functioning in daily living activities. Intact general cognitive function was defined as a Mini-Mental State Examination score >23 [29]. Objective cognitive decline was defined as having cognitive function more than 1.5 standard deviations lower than normal. Normal scores were taken from the Obu Study of Health Promotion for the Elderly (OSHPE) database of healthy individuals [27]. Cognitive function was also assessed in multiple domains using the National Center for Geriatrics and Gerontology Functional Assessment Tool [30]. Participants who suffered from cognitive decline in the memory domain were classified as aMCI, while those who did not were classified as naMCI.

### Gait measures

Participants wore the same type of appropriately sized shoes before each experiment. Participants were instructed to walk on a smooth 11-m horizontal walkway that had a 2-m buffer space at both ends for acceleration and deceleration. The time to walk 5 m to the mid-point of the walkway was measured, and gait speed was expressed in meters per second. Two gait experiments were performed sequentially: NW, in which participants walked at their preferred speed, was followed by DTW. Participants were instructed to walk while counting backward from 100 in DTW. This type of arithmetic task is commonly used in DTW investigations and its effects on gait have been confirmed in a meta-analysis [24].

### Cognitive function

Cognitive function was evaluated by comprehensive neuropsychological assessment and conducted by a well-trained speech therapist. Processing speed was assessed using a tablet version of the Symbol Digit Substitution Task (SDST) [30], based on the Symbol Digit Modalities Test [31]. The score is the number of correct answers chosen within 90 s. Executive function was evaluated using a tablet version of the Trail Making Test Part B (TMT-B, 15 stimuli) [30]. We recorded the amount of time it took to complete each task, and results were excluded from analysis if this time was greater than 90 seconds. Working memory was assessed using the digit span backward test, a subset of the Wechsler Adult Intelligence Scale III [32]. Verbal memory was assessed using the Rey Auditory Verbal Learning Test (RAVLT) [33]. Visual memory was examined using the visual reproduction subtest of the Wechsler Memory Scale-Revised (WMS-R) [34]. Better performance is represented by lower values in the TMT-B and higher values in the other tests.

### Other covariates

Age, sex, body mass index (weight/height^2^), and educational history were recorded as demographic data. Medical conditions and current medications were recorded. Apolipoprotein E (APOE) genotype was assessed using genomic DNA extracted from peripheral blood leukocytes or autopsy tissues using a standard method (SRL, Inc., Tokyo, Japan). The genotyped data were strictly controlled under condition of anonymity and blinded from the clinical information. Carrying ϵ4 is thought to be a strong factor related to deterioration of cognitive function in MCI participants [35]. To assess functional capacity, we used the Tokyo Metropolitan Institute of Gerontology Index of Competence [36] and activity level was measured using a life-space assessment [37].

### Statistical analysis

We compared participant characteristics between MCI subtypes (aMCI and naMCI) using an unpaired *t*-test for continuous variables or a chi-square test for categorical variables. Before examining the association between cognitive functions and gait variables, we first compared cognitive functions and gait variables between aMCI and naMCI groups. To compare cognitive function, we used a general linear model adjusted for age, which is thought to be a strong covariate, and participant characteristics that differed significantly between MCI subtypes. For gait variables, we used a repeated-measures analysis of variance (ANOVA) (adjusted for the same variables as above) to test for the main effects of MCI subtype (aMCI or naMCI) and walking condition (NW or DTW). To examine whether cognitive functions were independently associated with gait speed, we used a multivariable regression analysis adjusted for age, sex, body mass index, education, medication, life space, functional capacity, and APOE status as potential covariates. This adjusted model is conducted against gait speed under NW and DTW (model 1). Additionally, to clarify the association between cognitive function and gait speed under DTW, model 2 adjusted variables using model 1 added to gait speed in NW was conducted (model 2). All analyses were performed using commercially available software (JMP 9.0 J for Windows; SAS Institute Japan, Tokyo, Japan). Statistical significance was set at *p* < .05.

## Results

The 389 participants (52% women, mean age: 71.6 years) were classified as either aMCI (n = 191) or naMCI (n = 198). Table 1 summarizes the demographic data including educational history, current medication, functional capacity, life space, and status of APOE. The proportion of women was significantly different between MCI groups (aMCI: n = 79, 41%; naMCI: n = 124, 63%; *p* < .001), while other demographic variables were not. Therefore, when comparing cognitive functions between MCI groups, we adjusted for age and sex. RAVLT scores were lower in aMCI participants, while SDST scores were lower in naMCI participants (RAVLT: *p* < .001, SDST: *p* = .002). No significant differences between groups were found for the other cognitive functions. A repeated-measures ANOVA adjusted for age and sex showed that gait speed was affected by walking condition (NW vs. DTW: *p* = .042), but not by MCI group (naMCI vs. aMCI: *p* = .301).

**Table 1:** Subject characteristics

**Variables**	***M ± SD***
Age (years)	71.6 ± 4.9
Sex (women subjects (%))	203 (52)
Body mass index (kg/m^2^)	23.4 ± 2.9
Educational history (years)	11.0 ± 2.4
TMIG (score)	12.4 ± 1.1
Life-space assessment (score)	90.2 ± 15.7
Current medications (numbers)	2.2 ± 2.0
Type of MCI (amnestic MCI (%))	191 (49)
Status of apolipoprotein E (ϵ4 carrier (%))	76 (20)
Cognitive tests	
MMSE (score)	26.7 ± 1.9
SDST (score)	38.9 ± 7.4
TMT-B (s)	43.5 ± 16.7
Digit span backward (score)	5.1 ± 1.6
RAVLT-delay (score)	7.3 ± 3.4
Visual reproduction (score)	21.9 ± 8.8
Normal walking	
Gait speed (m/s)	1.36 ± 0.22
Dual-task walking	
Gait speed (m/s)	1.23 ± 0.32

*Note*: TMIG: Tokyo Metropolitan Institute of Gerontology Index of Competence. MCI: mild cognitive impairment. SDST: Symbol Digit Substitution Task. TMT-B: Trail Making Test Part B. RAVLT: Rey Auditory Verbal Learning Test

Values are mean ± SD or numbers (proportion).

**Figure 1:**
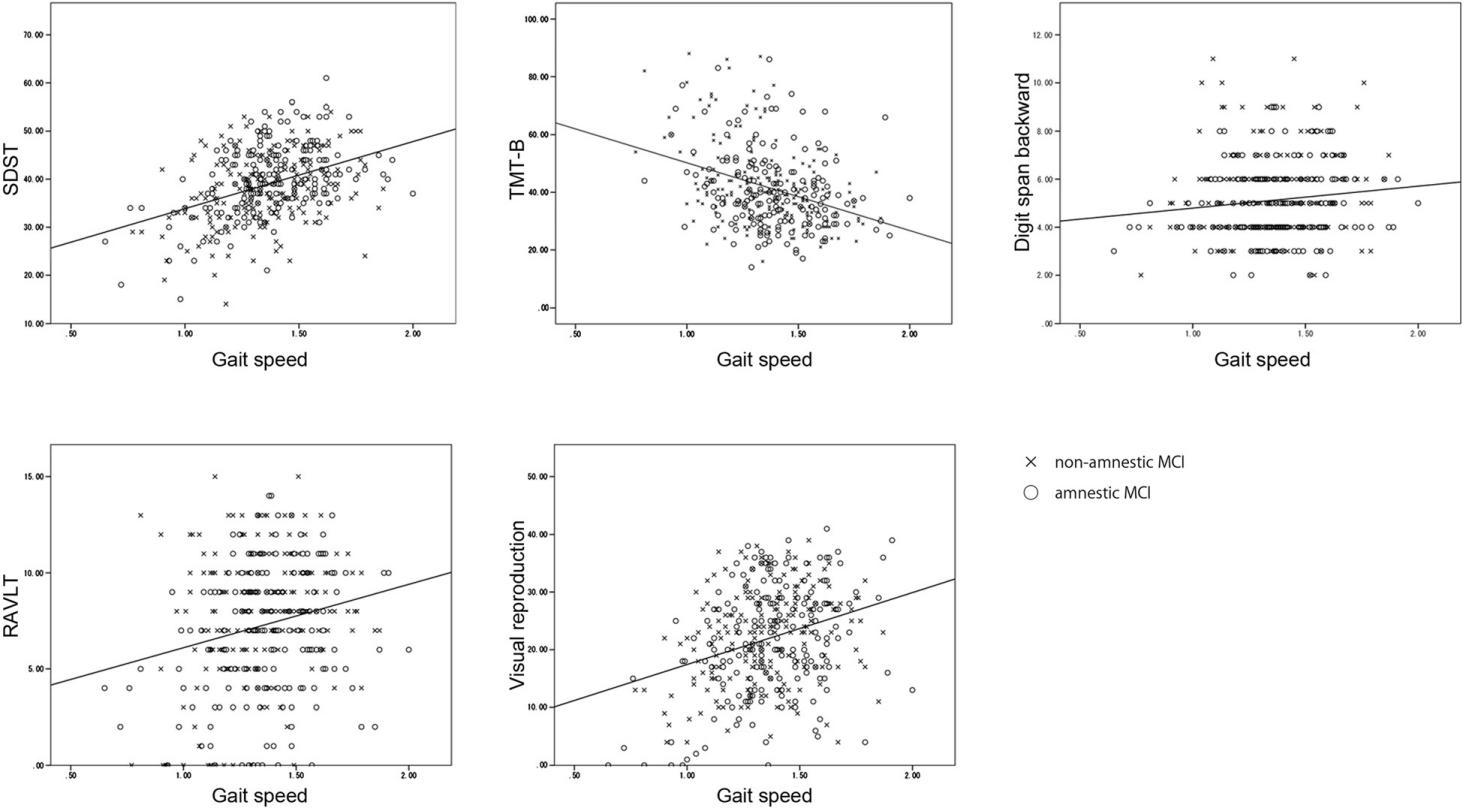
**Cognitive measurements and gait speed during normal walking.** Scatter plots showing scores of cognitive measurements and gait speed (m/s) during normal walking, grouped by MCI subtype. Lines indicate the resulting regression line for all participants.

**Figure 2:**
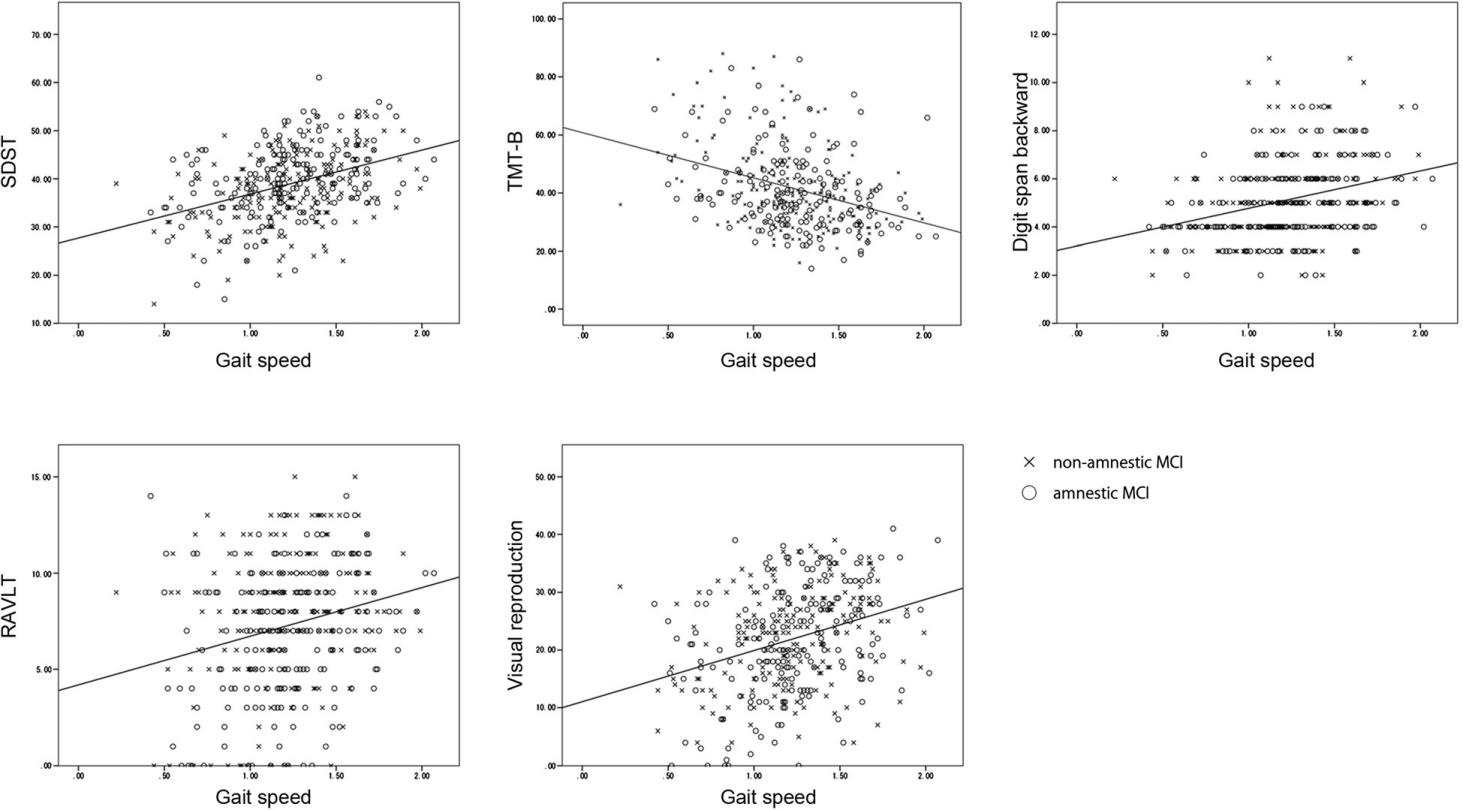
**Cognitive measurements and gait speed during dual-task walking.** Scatter plots showing scores of cognitive measurements and gait speed (m/s) during dual-task walking grouped by MCI subtype. Lines indicate the resulting regression line for all participants.

Simple correlation analysis showed a significant relationship between normal gait speed and all cognitive functions in all MCI participants (SDST: *r* = .406, *p* < .0001; TMT-B: *r* = -.375, *p* < .0001; digit span: *r* = .122, *p* = .0166; RAVLT: *r* = .209, *p* < .0001; visual reproduction: *r* = 0.306, *p* < .0001). DTW was also significantly associated with cognitive functions in all MCI participants (SDST: *r* = .395, *p* < .0001; TMT-B: *r* = -.373, *p* < .0001; digit span: *r* = .307, *p* < .0001; RAVLT: *r* = .238, *p* < .0001; visual reproduction: r = .325, *p* < .0001). Results from cognitive function tests are plotted against gait speed in Figure 1 (NW) and Figure 2 (DTW). A multivariate regression analysis adjusted for potential covariates was conducted and the results for gait variables during NW are summarized in Table 2. During NW, gait speed was associated with SDST scores in both MCI groups (aMCI: *p* = .003; naMCI: *p* = .009), with visual reproduction scores in aMCI participants (*p* = .037), and with TMT-B scores in naMCI participants (*p* = .025). Digit span and RAVLT were not significantly associated with gait speed during NW. Associations with gait speed during DTW are summarized in Table 3. Cognitive functions other than RAVLT correlated with gait speed in DTW even adjusted for normal gait speed in aMCI participants (all tests, *p* < .05), while only digit span did so in naMCI participants (*p* < .001).

**Table 2:** Multivariable regression results between cognitive function and gait speed during normal walking

		**Coefficients (SE)**		
**Cognitive measures**	**Cognitive domain**	**aMCI**	**naMCI**	**Total**
		**(n = 191)**	**(n = 198)**	**(n = 389)**
SDST	Processing speed	.216 (0.002)†	.202 (0.002)†	.209 (0.002)‡
TMT-B	Executive function	-.095 (0.001)	-.287 (0.001)‡	-.180 (0.001)‡
Digit span backward	Working memory	.013 (0.009)	.006 (0.009)	.006 (0.006)
RAVLT-delay	Verbal memory	.087 (0.004)	.025 (0.005)	.036 (0.003)
Visual reproduction	Visual memory	.142 (0.002)*	.066 (0.002)	.111 (0.012)*

*Note*: aMCI: amnestic mild cognitive impairment. naMCI: non-amnestic mild cognitive impairment. SDST: Symbol Digit Substitution Task. TMT-B: Trail Making Test Part B. RAVLT: Rey Auditory Verbal Learning Test.

Multivariable regression was adjusted for age, sex, body mass index, education, medication use, life space, functional capacity, and apolipoprotein E status.

*p < .05. †*p* < .01. ‡*p* < .001.

**Table 3:** Multivariable regression results between cognitive function and gait speed during dual-task walking

		**Coefficients (SE)**					
		**Model 1**			**Model 2**		
**Cognitive measures**	**Cognitive domain**	**aMCI**	**naMCI**	**Total**	**aMCI**	**naMCI**	**Total**
		**(n = 191)**	**(n = 198)**	**(n = 389)**	**(n = 191)**	**(n = 198)**	**(n = 389)**
SDST	Processing speed	.349 (0.004)‡	.214 (0.003)†	.269 (0.002)‡	.195 (0.003)†	.093 (0.003)	.134 (0.002)†
TMT-B	Executive function	-.203 (0.002)*	-.265 (0.002)†	-.237 (0.001)‡	-.148 (0.001)*	-.092 (0.001)	-.121 (0.001)†
Digit span backward	Working memory	.234 (0.015)†	.214 (0.013)†	.227 (0.010)‡	.226 (0.012)‡	.210 (0.009) ‡	.223 (0.007)‡
RAVLT-delay	Verbal memory	.174 (0.007)*	.047 (0.007)	.101 (0.005)	.120 (0.006)	.032 (0.005)	.079 (0.004)
Visual reproduction	Visual memory	.252 (0.003)‡	.109 (0.003)	.196 (0.002)‡	.166 (0.002)†	.068 (0.002)	.128 (0.002)†

*Note*: aMCI: amnestic mild cognitive impairment. naMCI: non-amnestic mild cognitive impairment. SDST: Symbol Digit Substitution Task. TMT B: Trail Making Test Part B. RAVLT: Rey Auditory Verbal Learning Test.

Model 1: Multivariable regression was adjusted for age, sex, body mass index, education, medication use, life space, functional capacity, and apolipoprotein E status. Model 2: adjusted for variables in model 1 and gait speed in normal walking.

*p < .05. †*p* < .01. ‡*p* < .001.

## Discussion

The results of this study indicate positive associations between cognitive functions and gait speed in MCI participants. The independent associations were revealed by a multivariate analysis adjusting for several potential confounding factors including the status of APOE. Processing speed and executive function correlated with gait speed during NW and DTW. Working memory was significantly associated with gait speed during DTW in both subtypes of MCI participants. Visual memory was also associated with gait speed in NW and DTW particularly in aMCI participants.

Our study showed that cognitive function in MCI participants is correlated with gait speed, and that this association differs depending on walking conditions (normal or dual-task). Indeed, some prospective studies have touched on this inter-relationship. Gait speed during NW has been shown to be related to cognitive decline [12], MCI [7], and risk of dementia [8], while impaired cognitive functions have been shown to be related to a decline in normal gait speed [9,11,13]. The majority of studies investigating this relationship have focused on normal gait speed and processing speed [12] or executive function [9,13], and have confirmed the relationship in older adults. Consistent with our results in MCI participants, McGough *et al*. have reported that physical performance is associated with executive function after adjusting for age, sex, and age-related factors in sedentary older adults with aMCI [38]. Here, we show that in addition to processing speed and executive function, gait speed during DTW is also associated with working memory in MCI participants, even after adjusting for normal gait speed. The effect of DTW on gait variables [24,25] and the requirement for executive function in older adults have been reported [21,22], and cognitive impairment (e.g., MCI) has been shown to have an impact on DTW performance. Montero-Odasso *et al*. [26] suggested that gait speed in MCI participants is related to working memory ability, and that the relationship is exaggerated during DTW. Our results partially agree with their study in that working memory was correlated with gait variables during DTW but not NW. Executive function is thought to be dominant in prefrontal lobe function. Processing speed has been reported to correspond to prefrontal lobe function, a region also thought to have a role in gait control [39]. Working memory systems are believed to be dominated and require similar neural resources in prefrontal cortex [40], although the resources for these functions are not fully identical. Our study supports the idea that prefrontal lobe function is required for gait in MCI participants.

The associations between cognitive function and gait speed differed depending on MCI subtype. To our knowledge, this is the first report showing that memory function requiring free recall is correlated with gait variables specifically in aMCI participants. Although a consensus regarding the relationship between memory function and gait ability has not been reached in studies of healthy older adults, our results are in line with prospective studies of healthy older adults [9,11]. Memory function in MCI, particularly aMCI, is a clinical signature of developing AD [2]. However, whether or not memory function relates to gait variables remains an open debate even when including studies using neuroimaging [41,42]. Unlike executive function, investigations focusing on the connection between memory and gait ability are few, and those that do have used variable measures of memory (e.g., verbal memory or visual memory). We examined verbal memory (RAVLT) and visual memory (visual reproduction subtest of the WMS-R) separately. Gait speed during both NW and DTW conditions correlated with visual memory functions in aMCI participants, while verbal memory function never correlated with gait speed. This result may reflect the fact that visual memory is required for visuospatial processing in addition to simple memory functions. In fact, cortical thickness [43] and gray matter [41] in visual processing regions are correlated with gait variables during NW. Further study is required to clarify the relationship between memory function and gait performance.

Our study had several strengths and limitations. We used a large cohort with a sufficient sample size. Additionally, our analysis included adjustments for several potential covariates, such as the status of APOE, that affect not only pathogenesis (e.g., Aβ aggregation or neural toxicity) [44] but cognitive decline [35]. However, some limitations must be noted. Because a cross-sectional design was used, the causal relationship between cognitive function and gait is still unclear in people with MCI. Further prospective studies are required to address this issue. Additionally, the type and/or difficulty of the cognitive task used for DTW could have affected the results. While the mental tracking task we adopted (counting backwards) is widely used, the effects of dual tasking on gait may depend on the cognitive task [24]. Hence, DTW using other types of cognitive tasks (e.g., verbal fluency) should be investigated. Finally, neuroimaging methods have recently been used to clarify the cortical control of gait. Further evidence using imaging techniques should be gathered to clarify the association between cognitive function and gait ability under varied conditions.

## Conclusion

Successful DTW for those with MCI may require adequate cognitive function, processing speed, executive function, working memory and visual memory. The association between cognitive functions and gait variables partially depends on the MCI subtype. Gait speed in both NW and DTW are associated with memory performance particularly in MCI participants whose memory performance has declined (aMCI) compared with those with relatively intact memory functions (naMCI). Further studies are needed to clarify the effects of cognitive function on gait in MCI participants.

## Competing interests

The authors declare that they have no competing interests.

## Authors’ contributions

TD substantially contributed to the conception of the methods used, participant recruitment, analysis and writing the manuscript. HS and HM made substantial contributions to conception and design, participant recruitment, and writing the manuscript. KT and KU were involved in the acquisition, analysis and interpretation of data. YA contributed to the acquisition of data. TS made substantial contributions to the conception and design and writing the manuscript. All authors read and approved the final manuscript.

## Pre-publication history

The pre-publication history for this paper can be accessed here:

http://www.biomedcentral.com/1471-2377/14/67/prepub
